# Construction and validation of a prediction model for malignant pulmonary nodules based on imaging, demographic, and laboratory features

**DOI:** 10.3389/fonc.2026.1774574

**Published:** 2026-07-27

**Authors:** Yuxuan Qi

**Affiliations:** Department of Radiology, The Traditional Chinese Medical Hospital of Fengtai District, Beijing, China

**Keywords:** imaging features, integrated diagnosis, non-contrast CT, predictive model, solitary pulmonary nodule

## Abstract

**Introduction:**

This study aimed to explore the risk factors for malignant solitary pulmonary nodules (SPNs) and to develop an early integrated diagnostic model by analyzing patients’ demographic data, clinical laboratory indicators, and imaging findings.

**Methods:**

This retrospective study analyzed the medical records of 516 patients with a solitary pulmonary nodule(SPN) between December 2022 and August 2025. The cohort consisted of 402 patients with benign SPN and 114 with malignant SPN. Baseline characteristics were analyzed using SPSS 25 (version 25), and all subsequent statistical analyzes were performed using R 4.1.0. Variables were treated as categorical, with a two-sided p ≤ 0.05 considered statistically significant. Independent predictors of malignant SPN were identified using logistic regression and least absolute shrinkage and selection operator (LASSO) regression models. Model performance was evaluated and compared using the area under the receiver operating characteristic curve (ROC-AUC), calibration curves, and decision curve analysis (DCA). Based on a comprehensive comparison, the final predictive model was selected and presented as a nomogram to facilitate individualized clinical decision-making.

**Results:**

Logistic regression analysis identified five independent predictors for the benign or malignant status of pulmonary nodules:age (p = 0.04), smoking history (p = 0.02), nodule diameter (p = 0.01), lobulation (p = 0.02), and spiculation (p = 0.02). The least absolute shrinkage and selection operator (LASSO) regression model indicated that spiculation, nodule diameter, carcinoembryonic antigen, and smoking history possessed independent predictive value. Based on a comparison of predictive performance, the logistic regression model (both univariate and multivariate) demonstrated superior discrimination and calibration compared to the LASSO regression model. Consequently, a visual nomogram was developed based on the results of the logistic regression analysis.

**Conclusion:**

This study employed dual modeling with logistic regression and LASSO regression. The analysis identified five independent predictors for malignant SPN: age, smoking history, nodule diameter, lobulation, and spiculation. In comparison, the logistic regression model demonstrated superior discrimination and calibration. Based on these findings, a visual nomogram was developed to facilitate clinical application.

## 1 Introduction

This study was designed to develop and validate a predictive model for lung nodule malignancy in adults undergoing low-dose CT screening, by integrating CT features with demographic and laboratory indicators. The target population comprises individuals aged 40–75 years with incidentally detected or screening-identified pulmonary nodules (5–30 mm), a group at elevated risk for lung cancer yet often subject to invasive procedures or delayed diagnosis due to indeterminate nodule assessment. We employed least absolute shrinkage and selection operator (LASSO) regression for initial feature screening, followed by multivariable logistic regression with backward elimination to construct the final model, ensuring parsimony and clinical interpretability. The model aims to improve the accuracy of early lung cancer diagnosis, thereby offering substantial clinical benefit to patients with early-stage disease. Decision curve analysis was performed to quantify net benefit across threshold probabilities, and net reclassification improvement was calculated to evaluate incremental value over existing models. Internal validation was conducted using 10-fold cross-validation and bootstrapping (1000 resamples) to assess model stability and optimism-corrected performance. While external validation using independent multicenter data is recognized as essential for confirming generalizability, this was not feasible within the current study due to data availability constraints; thus, the present findings should be interpreted as development and internal validation, with external validation planned as the immediate next step to support broader clinical implementation. The model’s novelty lies in its incorporation of systemic inflammatory markers and metabolic indicators alongside radiomic CT features, addressing a gap in existing lung nodule risk models that rely predominantly on morphological characteristics or limited clinical variables.

## 2 Materials and methods

This retrospective, single-center study was conducted at the Traditional Chinese Medical Hospital of Fengtai District, Beijing. We acknowledge that the single-center retrospective design inherently carries potential selection bias, including referral bias and spectrum bias, which may limit the generalizability of findings to other clinical settings. This limitation is discussed in detail in the Discussion section.

Between December 2022 and August 2025, 516 patients with solitary pulmonary nodules (SPN) were consecutively enrolled. The cohort consisted of 402 patients with benign SPN and 114 with malignant SPN, reflecting the natural prevalence imbalance observed in clinical practice. There were 281 males and 235 females, with a mean age of 59.00 ± 7.92 years. All patients were aged over 18 years and had been screened to exclude extra-pulmonary malignancies. Each patient had a definitive pathological diagnosis confirmed by postoperative or biopsy examination. The clinical characteristics of all 516 patients are summarized in **Table 1**.

**Table 1:** Baseline characteristics.

Features	Derivation cohort (n=378)	Internal validation cohort (n=138)
Pathological results		
Benign	300 (79.4%)	102 (73.8%)
Malignant	78 (20.6%)	36 (26.2%)
Age (years)		
≤40	119 (31.5%)	46 (33.3%)
40-50	102 (19.8%)	30 (21.7%)
50-60	60 (15.9%)	23 (16.7%)
60-70	40 (10.6%)	17 (12.3%)
≥70	87 (23.0%)	22 (15.9%)
Gender		
Male	171 (45.2%)	64 (46.4%)
Female	207 (54.8%)	74 (53.6%)
Smoking history		
Yes	149 (39.4%)	52 (37.7%)
No	229 (60.6%)	86 (62.3%)
Occupational exposure		
Yes	159 (42.1%)	58 (42.0%)
No	219 (57.9%)	80 (58.8%)
Family history of genetic diseases		
Yes	171 (42.1%)	63 (45.7%)
No	207 (54.8%)	75 (54.3%)
Lobulation		
Yes	103 (27.2%)	39 (28.3%)
No	275 (72.8%)	99 (71.7%)
Spiculation		
Yes	64 (27.8%)	33 (23.9%)
No	273 (72.2%)	105 (76.1%)
Cavitation		
Yes	60 (15.9%)	24 (17.4%)
No	318 (84.1%)	114 (82.6%)
Vascular convergence		
Yes	105 (27.8%)	40 (29.0%)
No	273 (72.2%)	98 (71.0%)
Pleural indentation		
Yes	110 (29.1%)	42 (30.4%)
No	268 (70.9%)	96 (69.6%)
Nodule diameter (mm)		
≤5	103 (27.2%)	35 (25.4%)
5-8	151 (39.9%)	56 (40.6%)
8-15	62 (16.4%)	21 (15.2%)
≥15	62 (16.4%)	26 (18.8%)
CEA (ng/ml)		
≤5	144 (38.1%)	56 (40.6%)
5-10	125 (33.1%)	39 (28.3%)
10-20	60 (15.9%)	23 (16.7%)
≥20	49 (13.0%)	20 (14.5%)
CYFRA21-1 (ng/ml)		
≤3.3	159 (42.1%)	59 (42.8%)
3.3-7.5	162 (42.9%)	57 (41.3%)
≥7.5	57 (15.1%)	22 (15.9%)
CA-125 (U/ml)		
≤35	177 (46.8%)	62 (44.9%)
35-53	78 (20.6%)	29 (21.0%)
53-100	69 (18.3%)	29 (21.0%)
≥100	54 (14.3%)	18 (13.0%)
PLR		
≤3	228 (60.2%)	83 (60.1%)
3-5	96 (25.4%)	36 (26.1%)
≥5	54 (14.3%)	19 (13.8%)
Albumin (g/dl)		
≥40	159 (42.1%)	56 (40.6%)
35-40	96 (25.4%)	35 (25.4%)
30-35	81 (21.4%)	29 (21.0%)
≤30	42 (11.1%)	18 (13.0%)
Procalcitonin (ng/ml)		
≤0.05	219 (57.9%)	81 (58.8%)
0.05-0.5	132 (34.9%)	50 (36.3%)
≥0.5	27 (7.1%)	7 (4.9%)

CEA, carcinoembryonic antigen; PLR, platelet-to-lymphocyte ratio.

A database was established using patient admission records to collate information and routine examination data, encompassing radiological findings, demographic baseline indicators, pathological results, and clinical laboratory metrics.

A total of 516 patients with SPN were randomly allocated to a model derivation group (n = 378) and a model validation group (n = 138) in a 7:3 ratio. Given the inherent class imbalance (benign:malignant = 3.5:1), we employed random stratified sampling to ensure proportional representation of benign and malignant cases in both derivation and validation sets. Sensitivity analyzes were performed to assess model robustness across different prevalence scenarios, as detailed in the Statistical Methods section.

Based on pathological analysis, the cases were categorized as either benign SPN (n = 402) or malignant SPN (n = 114). The benign group comprised 77 cases of interstitial lung disease, 82 hamartomas, 86 fibrotic nodules, 83 cases of organizing pneumonia, 55 inflammatory pseudotumors, and 19 lung abscesses. The malignant group included 15 small cell carcinomas, 72 adenocarcinomas, and 27 squamous cell carcinomas. All participants were under continuous follow-up. The study protocol was approved by the hospital’s Ethics Committee (Ethics Review Number: KJK-2025009).

### 2.1 Inclusion criteria

1. A non−contrast chest CT scan was performed on a 64−slice scanner (General Electric, GE) using a standardized protocol in the supine position under breath−hold. 2. All patients had a definitive pathological diagnosis confirmed by surgical resection or biopsy. 3. The SPN was newly detected, with no prior history of treatment.

### 2.2 Exclusion criteria

1. Presence of concurrent malignant tumors in other organs. 2. Age younger than 18 years. 3. Previous diagnosis of pulmonary tuberculosis, pneumonia, lung cancer, or other pulmonary diseases, or with previously confirmed pulmonary nodules. 4. Pulmonary nodule diameter exceeding 3 cm or presenting with signs of lymph−node involvement. 5. Incomplete demographic, pathological, or imaging data.

### 2.3 CT acquisition protocol

A 64-slice CT scanner (General Electric, GE, USA) was used with the following parameters: detector configuration, 64 × 0.5 mm; tube current, 250 mA; tube voltage, 120 kV; slice spacing and slice thickness, 5 mm each. All patients were scanned in the supine position under breath-hold. The scan range extended from the thoracic inlet to the lung base.

Non-contrast CT was selected as the imaging modality for several reasons: (1) it is the standard protocol for initial lung cancer screening and incidental nodule detection in our institution and nationwide screening programs, maximizing clinical applicability; (2) it avoids iodinated contrast exposure, particularly relevant for patients with renal impairment or contrast allergies who require nodule evaluation; (3) morphological features predictive of malignancy (lobulation, spiculation, pleural indentation, calcification patterns) are equally well-visualized on non-contrast images; and (4) it reduces examination cost and time, facilitating broader implementation. We acknowledge that contrast-enhanced CT provides additional vascular information; however, our model focuses on accessible, low-risk screening parameters.

### 2.4 Radiological feature extraction

The imaging studies were independently reviewed by a panel comprising two senior radiologists (with 15 and 12 years of thoracic imaging experience, respectively). They delineated regions of interest (ROIs) and performed precise measurements, culminating in the issuance of comprehensive diagnostic reports.

#### 2.4.1 Interobserver variability assessment

To evaluate reproducibility, 50 randomly selected cases (approximately 10% of the cohort) were independently assessed by both radiologists. Discrepancies were resolved by consensus discussion with a third senior radiologist.

CT imaging data were systematically collected from patients with SPN. The process was standardized using uniform equipment, procedures, and parameters to ensure consistency and reliability.

**Figure 1:**
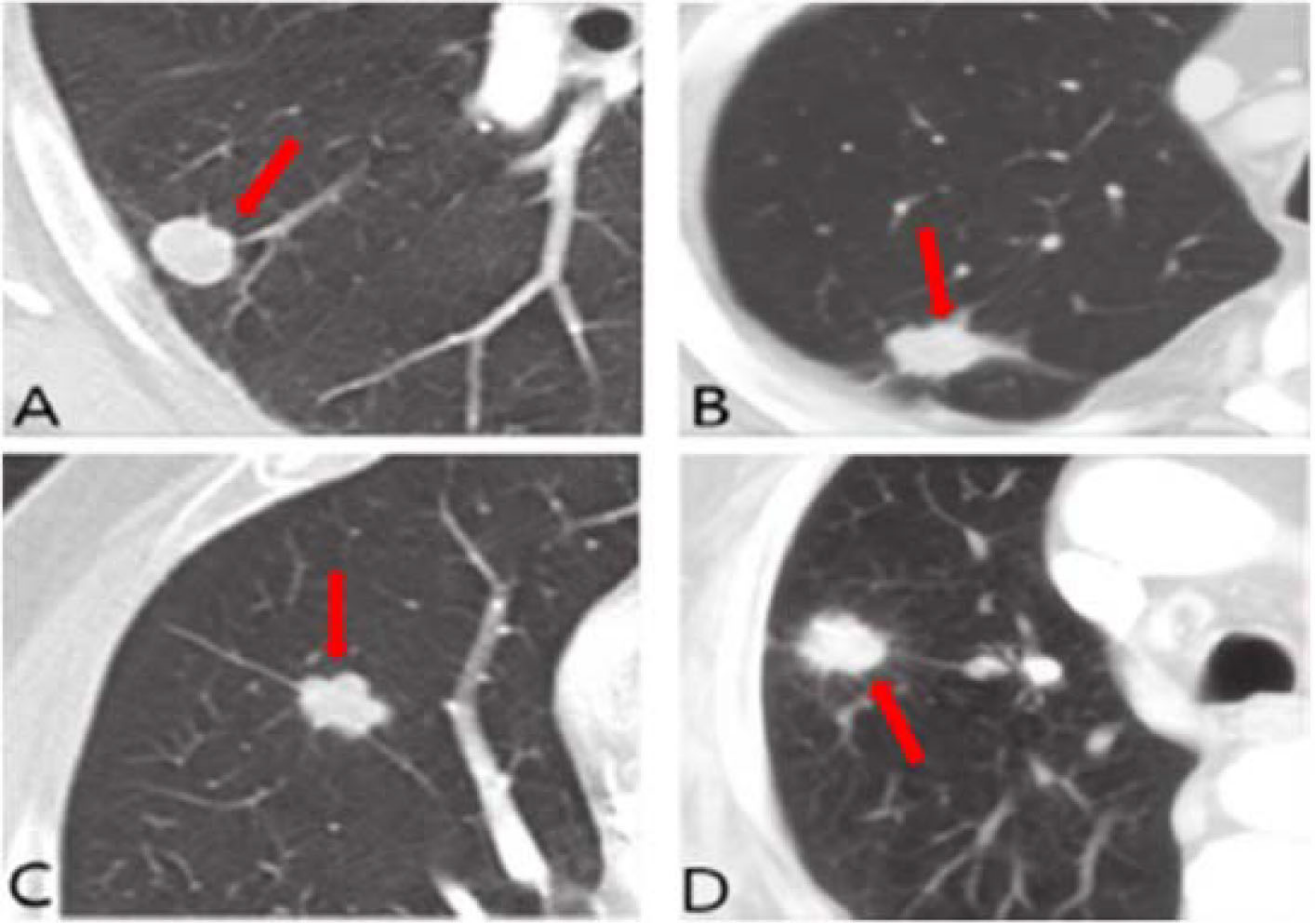
Representative CT imaging characteristics of patients with SPN. Computed tomography images of four patients with solitary pulmonary nodules (SPN): Patient 1, male, 60 years old; Patient 2, female, 58 years old; Patient 3, female, 59 years old; Patient 4, male, 65 years old. Histopathological diagnoses: **(A)** benign nodule; **(B)** small cell carcinoma; **(C, D)** lung adenocarcinoma.

**Figure 2:**
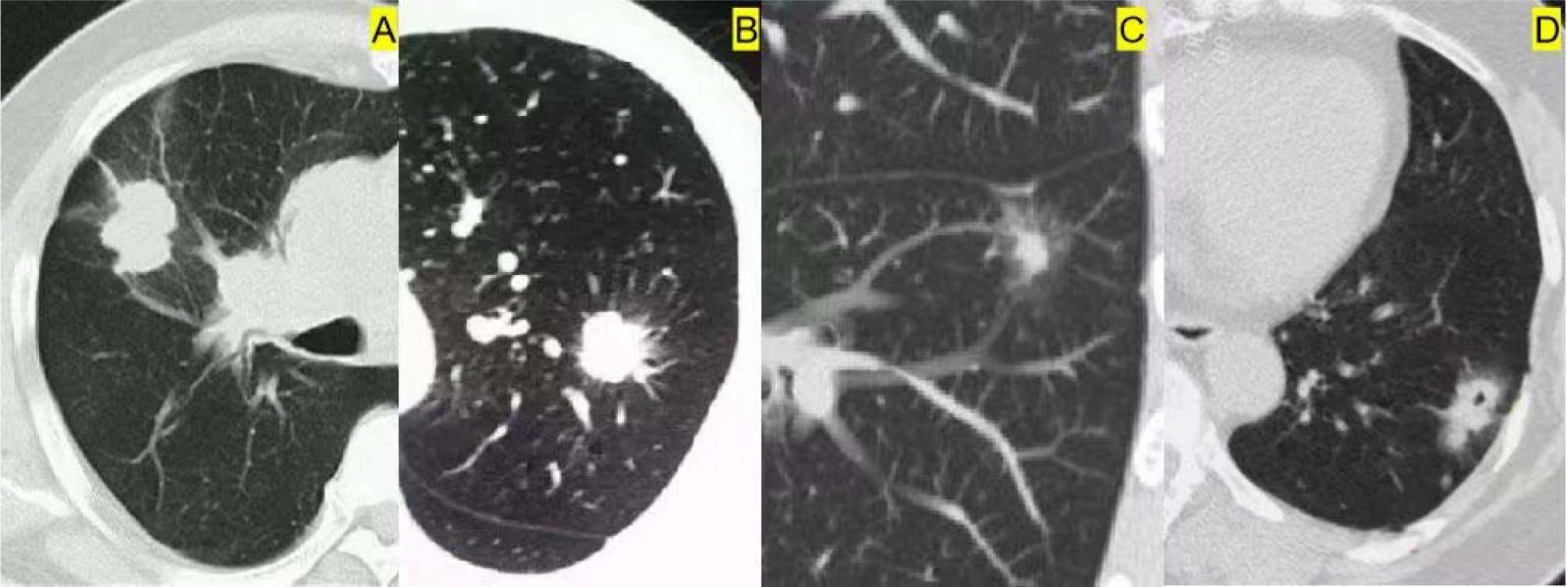
Schematic diagram of imaging features. Computed tomography imaging features of solitary pulmonary nodules: **(A)** lobulated sign; **(B)** spiculation; **(C)** vessel convergence sign; **(D)** cavitation sign.

From a morphological perspective, a detailed analysis of nodule edge characteristics was conducted. This included assessment for lobulation, edge smoothness, and spiculation. Concurrently, the maximum nodule diameter was measured, and surrounding features such as vascular convergence and pleural indentation were evaluated. The internal characteristics of the nodules were also assessed, including the presence or absence of cavitation and calcification (seen in **Figures 1**, **2**).

### 2.5 Statistical methods

Baseline patient characteristics were analyzed using SPSS (version 25.0). All variables were treated as categorical and are presented as frequencies and proportions. Subsequent statistical analyzes were performed using R (version 4.1.0), with a two-sided p-value ≤ 0.05 considered statistically significant.

#### 2.5.1 Missing data handling

Missing data were assessed for pattern (missing completely at random, missing at random, or missing not at random). Variables with >20% missing data were excluded. For remaining missing values, multiple imputation by chained equations (MICE) was performed with 20 imputed datasets, and results were pooled using Rubin’s rules.

#### 2.5.2 Model development and validation

Univariate and multivariate logistic regression analyzes were conducted to identify predictors of malignant SPN. Least absolute shrinkage and selection operator (LASSO) regression was additionally employed for variable selection to address high dimensionality and multicollinearity, with optimal lambda determined by 10-fold cross-validation.

#### 2.5.3 Overfitting prevention

To prevent overfitting, we employed: (1) LASSO regularization for feature selection; (2) strict sample size criteria (events per variable >10); (3) 10-fold cross-validation during model development; (4) separate temporal validation using the 7:3 split; and (5) bootstrapping (1000 resamples) for optimism-corrected performance estimates. The final model was selected based on the minimum optimism-corrected AIC.

#### 2.5.4 Model performance evaluation

Receiver operating characteristic (ROC) curves were generated and the area under the curve (AUC) was calculated for both the derivation and validation datasets. Calibration curves were constructed to assess the agreement between predicted and observed probabilities, with optimal calibration indicated by proximity to the 45-degree diagonal. Decision curve analysis (DCA) was further used to evaluate the clinical net benefit across different threshold probabilities.

#### 2.5.5 Sensitivity analysis for class imbalance

Given the benign-to-malignant ratio of 3.5:1, we performed sensitivity analyzes using: (1) random undersampling of the majority class; (2) synthetic minority oversampling technique (SMOTE); and (3) cost-sensitive learning with class weights. Model performance metrics were compared across these scenarios to assess robustness to prevalence imbalance.

Nomogram Construction: Finally, the optimal predictive model was visualized as a nomogram using the “rms” package in R, offering an intuitive tool to support clinical decision-making.

## 3 Results

### 3.1 Baseline clinical data characteristics

A total of 516 patients were included, with 378 (73.2%) allocated to the derivation cohort and 138 (26.8%) to the internal validation cohort. The derivation cohort contained 78 malignant cases, compared with 36 in the internal validation cohort. Baseline characteristics were well−balanced between cohorts. Regarding age, patients aged ≤40 years accounted for 31.5% in the derivation cohort and 33.3% in the internal validation cohort, while those aged ≥70 years represented 23.0% and 15.9%, respectively. Gender distribution was similar (approximately 45.2% vs. 46.4% male). The prevalence of risk factors, including smoking history (39.4% vs. 37.7%) and occupational exposure (42.1% vs. 42.0%), was comparable. Radiological features—lobulation, spiculation, and vascular convergence—exhibited closely matched frequencies. Tumor−marker analysis showed that CEA >5 ng/ml was present in approximately 60% of patients in both cohorts, and CYFRA21−1 >3.3 ng/ml in about 57%. For inflammatory indicators, PLR >3 was observed in roughly 40% of patients, and procalcitonin >0.05 ng/ml was present in approximately 40% of both cohorts (seen **Table 1**).

Among the 25 variables assessed, 8 variables had >20% missing data and were excluded from further analysis Little’s MCAR test indicated that missing data were missing completely at random. Multiple imputation by chained equations (MICE) with 20 imputed datasets was applied to the remaining variables. The pooled results from imputed data were consistent with those from complete-case analysis. These data indicated that the internal validation cohort was well-matched with the derivation cohort in terms of demographic characteristics, clinical indicators and radiological manifestation, providing a reliable basis for model validation.

### 3.2 Results of univariate and multivariate logistic regression analyzes

In this study, univariate and multivariate logistic regression analyzes were conducted on 378 patients to systematically identify predictors of pulmonary nodule malignancy. Univariate analysis revealed several statistically significant factors, including clinical indicators such as age (p = 0.04), smoking history (p = 0.02), occupational exposure (p = 0.05), nodule diameter (p = 0.01), carcinoembryonic antigen (p = 0.02), and CA-125 (p = 0.02), as well as radiological features including lobulated sign (p = 0.01) and spiculation (p = 0.01). Subsequent multivariate analysis identified five independent predictors: age (p = 0.04), smoking history (p = 0.02), nodule diameter (p = 0.01), lobulated sign (p = 0.02), and spiculation (p = 0.02). These findings indicate that a combined assessment of clinical characteristics, tumor markers, and radiological features holds significant value in differentiating benign from malignant pulmonary nodules, thereby providing an objective basis for clinical decision-making (see **Table 2**).

**Table 2:** The results of univariate/multivariate logistic regression.

Features	Univariate logistic		Multivariate logistic	
	95% CI	P values	95% CI	P values
Age				
≤40	Reference	Reference	Reference	Reference
40-50	0.44-4.87	0.16	0.52-2.54	0.25
50-60	0.61-2.92	0.33	0.65-1.98	0.38
60-70	1.42-1.71	0.04	0.98-1.66	0.15
≥70	1.98-2.98	0.02	1.12-2.45	0.04
Gender				
male	Reference	Reference		
female	0.42-2.39	0.77		
Smoking history				
Yes	Reference	Reference	Reference	Reference
No	0.33-0.45	0.02	0.45-0.66	0.02
Occupational exposure				
Yes	Reference	Reference		
No	0.34-0.99	0.05		
Family history of genetic diseases				
Yes	Reference	Reference		
No	0.45-2.17	0.87		
Nodule diameter (mm)				
≤5	Reference	Reference	Reference	Reference
5-8	0.41-2.05	0.51	0.65-1.85	0.62
8-15	1.22-2.36	0.02	1.23-2.52	0.02
≥5	2.44-4.21	0.01	1.22-2.94	0.01
CEA (ng/ml)				
≤5	Reference	Reference		
5-10	0.45-1.38	0.25		
10-20	1.34-1.62	0.02		
≥20	1.23-1.44	0.03		
CYFRA21-1(ng/ml)				
≤3.3	Reference	Reference		
3.3-7.5	0.42-6.80	0.39		
≥7.5	0.24-4.08	0.99		
CA-125(U/ml)				
≤35	Reference	Reference		
35-53	1.35-1.76	0.02		
53-100	0.48-1.67	0.56		
≥100	0.07-1.17	0.29		
PLR				
≤3	Reference	Reference		
3-5	0.18-2.13	0.45		
≥5	0.25-3.54	0.94		
Albumin(g/dl)				
≥40	Reference	Reference		
35-40	0.16-2.01	0.41		
30-35	0.18-2.51	0.55		
≤30	0.18-2.55	0.61		
Procalcitonin(ng/ml)				
≤0.05	Reference	Reference		
0.05-0.5	0.11-1.87	0.45		
≥0.5	0.08-1.72	0.37		
Lobulation				
Yes	Reference	Reference	Reference	Reference
No	0.10-0.50	0.01	0.11-0.58	0.02
Spiculation				
Yes	Reference	Reference	Reference	Reference
No	0.03-0.48	0.01	0.15-0.55	0.02
Cavitation				
Yes	Reference	Reference		
No	0.54-1.22	0.08		
Vascular convergence				
Yes	Reference	Reference		
No	0.23-1.30	0.17		
Pleural indentation				
Yes	Reference	Reference		
No	0.22-1.24	0.15		

CEA, Carcinoembryonic Antigen; PLR, Platelet-to-Lymphocyte Ratio.

### 3.3 Results of LASSO regression analysis

**Figure 3:**
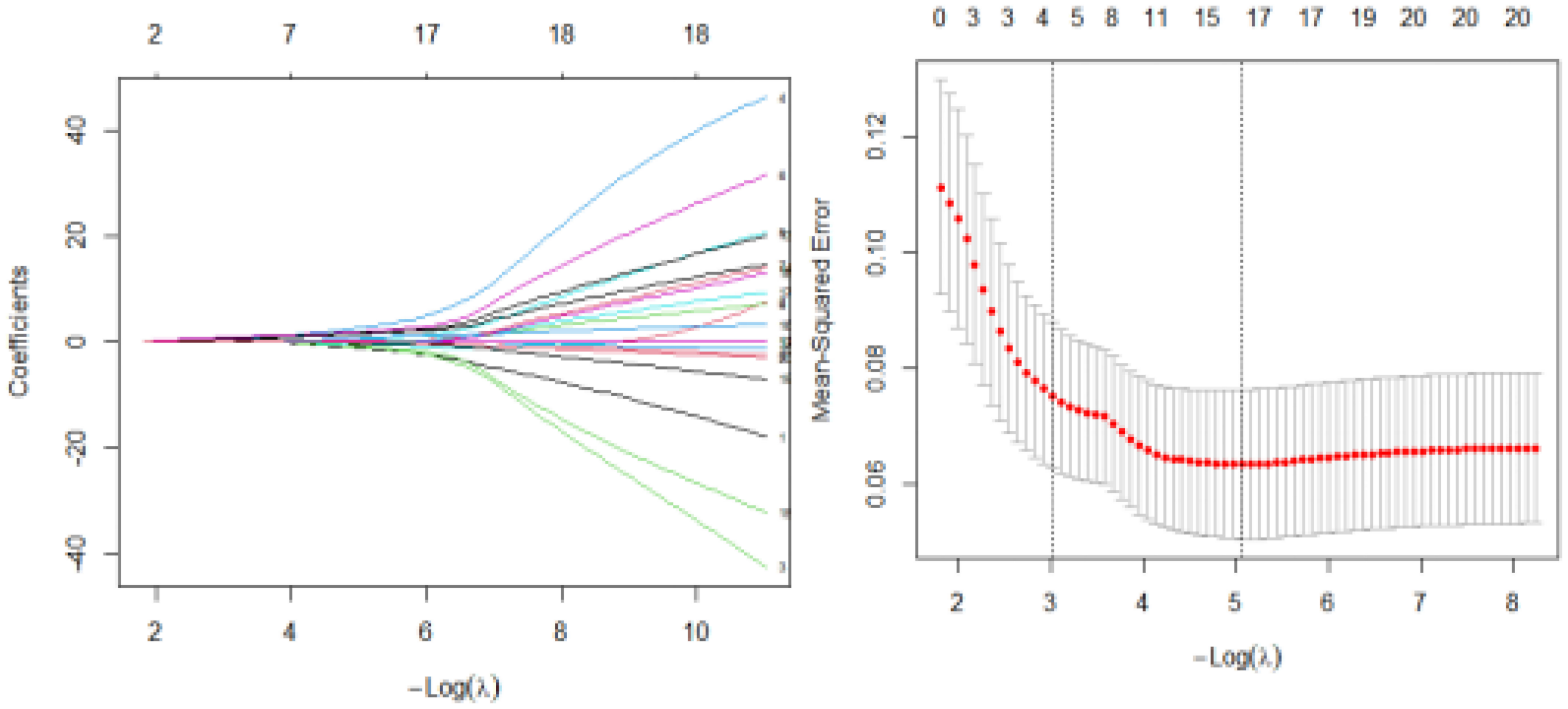
LASSO regression path plot.

LASSO regression was employed to select predictive features. The L1 regularization penalty term (with λ = 0.0063 determined through 10−fold cross−validation) enabled feature selection while maintaining model predictive accuracy and addressing potential multicollinearity. The analysis identified spiculation, nodule diameter, carcinoembryonic antigen, and smoking history as independent predictors (**Figure 3**).

### 3.4 Comparison of the predictive performance of the two models

**Figure 4:**
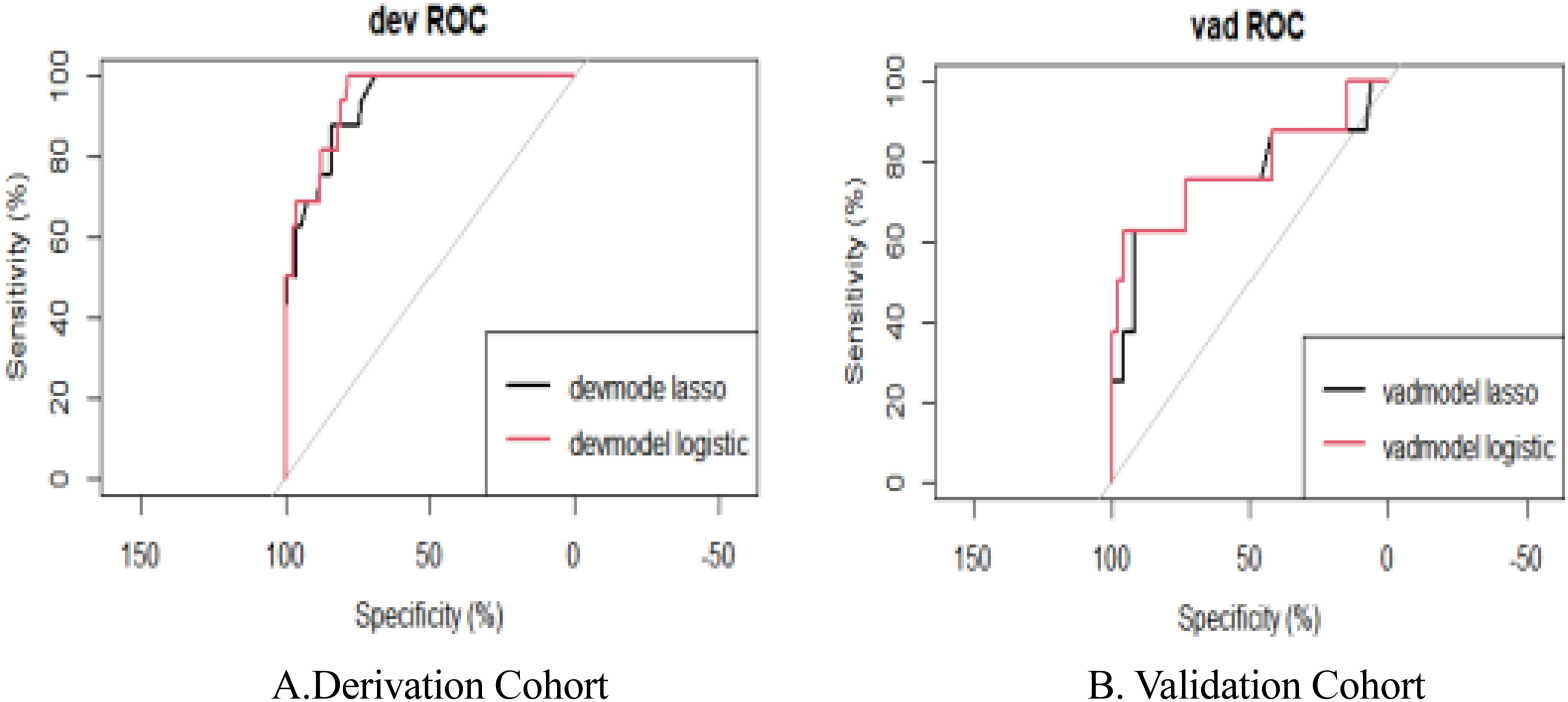
ROC curves of the two prediction models in the derivation and internal validation cohorts.

ROC curves were constructed for both the derivation cohort(N = 378) and the internal validation cohort (N = 138) (**Figure 4**). To evaluate model performance, the area under the curve (AUC), sensitivity, and specificity were calculated for each model in both cohorts. In the derivation cohort, the logistic regression model achieved an AUC of 0.942, a sensitivity of 0.782, and a specificity of 0.922. In comparison, the LASSO regression model yielded an AUC of 0.911, a sensitivity of 0.837, and a specificity of 0.822. In the internal validation cohort, the logistic regression model demonstrated an AUC of 0.779, a sensitivity of 0.958, and a specificity of 0.625, whereas the LASSO regression model showed an AUC of 0.754, a sensitivity of 0.917, and a specificity of 0.664. Overall, the logistic regression model consistently outperformed the LASSO regression model in predictive efficacy across both cohorts (**Figure 4**).

### 3.5 Clinical calibration curves for the two prediction models

**Figure 5:**
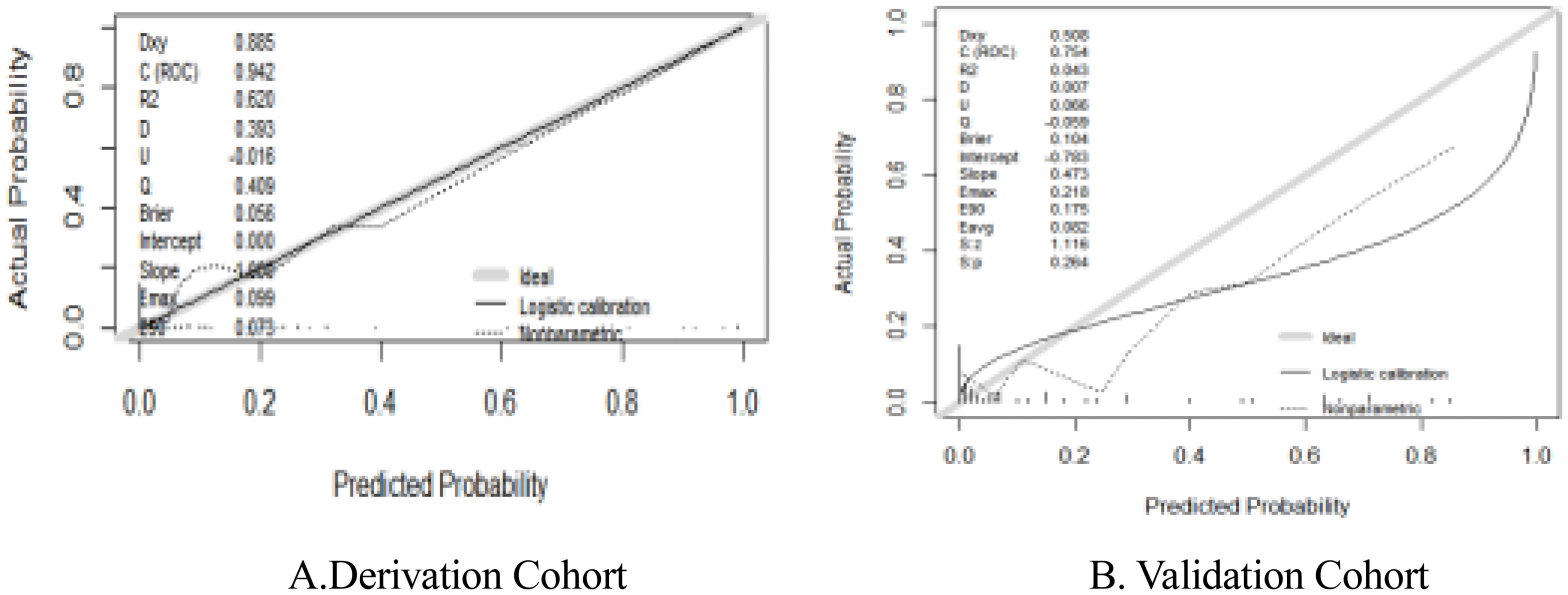
Clinical calibration curves of the univariate and multivariate regression models. **(A)** Derivation cohort; **(B)** validation cohort.

**Figure 6:**
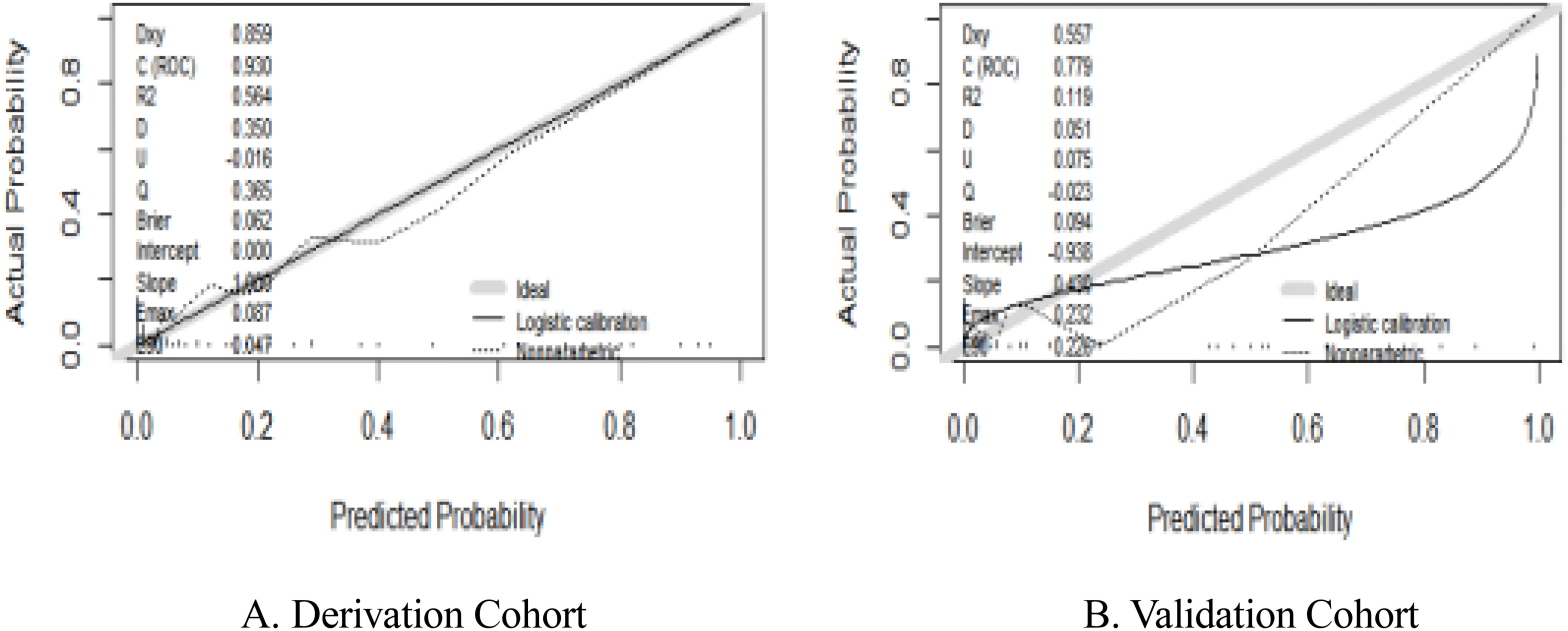
Clinical calibration curves of the Lasso regression model. **(A)** Derivation cohort; **(B)** validation cohort.

Calibration curves were plotted to assess the agreement between predicted and observed outcomes for malignant pulmonary nodules. In these plots, the x-axis represents the model-predicted probability of malignancy, and the y-axis represents the actual observed probability. A curve closer to the diagonal reference line indicates better calibration performance within the cohort (**Figures 5**, **6**).

Both prediction models showed good consistency in the derivation and validation sets, especially in the derivation set. In addition, there was a certain deviation between the predicted values and observed values of the two models in the external validation set, which requires further improvement and validation in a larger sample case cohort dataset.

### 3.6 Clinical decision curve analysis of the prediction models

**Figure 7:**
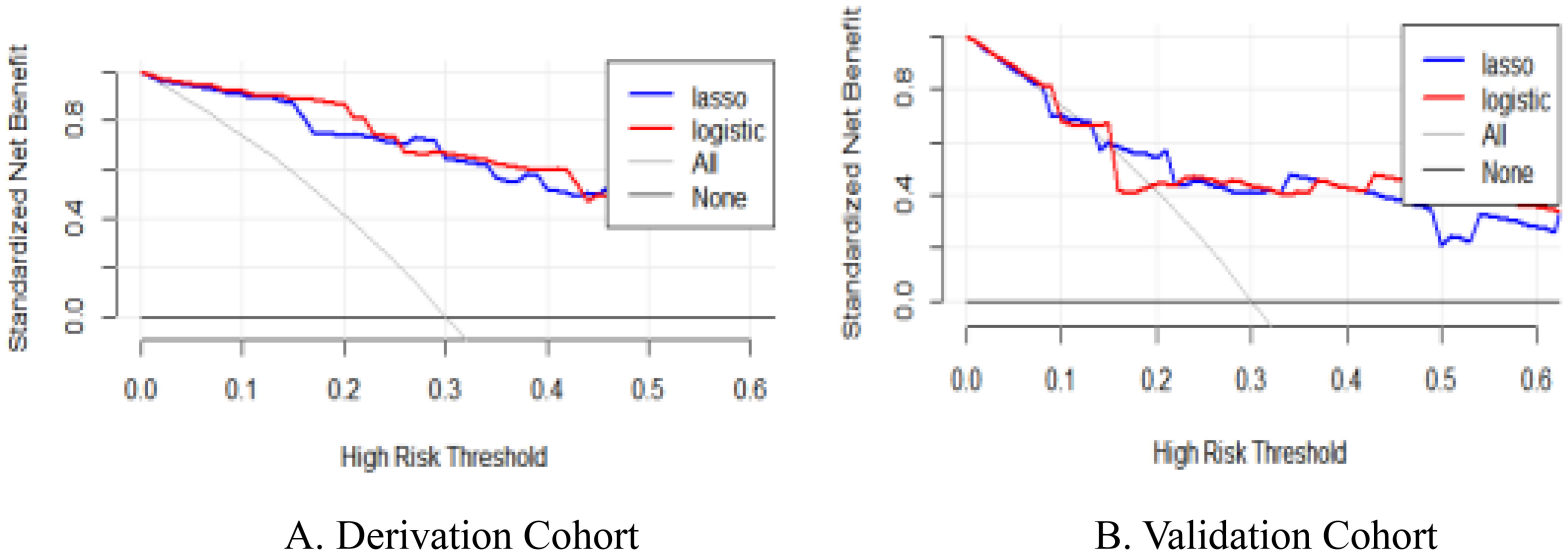
Clinical decision curves of the two prediction models. **(A)** Derivation cohort; **(B)** validation cohort.

Decision curve analysis(DCA) was performed to evaluate the clinical utility of the two models. In the derivation cohort, both models provided significant net clinical benefit across a range of probability thresholds. In the internal validation cohort, the analysis demonstrated that the logistic regression model offered a significant net benefit when the predicted probability threshold exceeded 20%, while the LASSO regression model provided a significant net benefit at a threshold above 15% (**Figure 7**).

### 3.7 Development of a visual predictive nomogram

**Figure 8:**
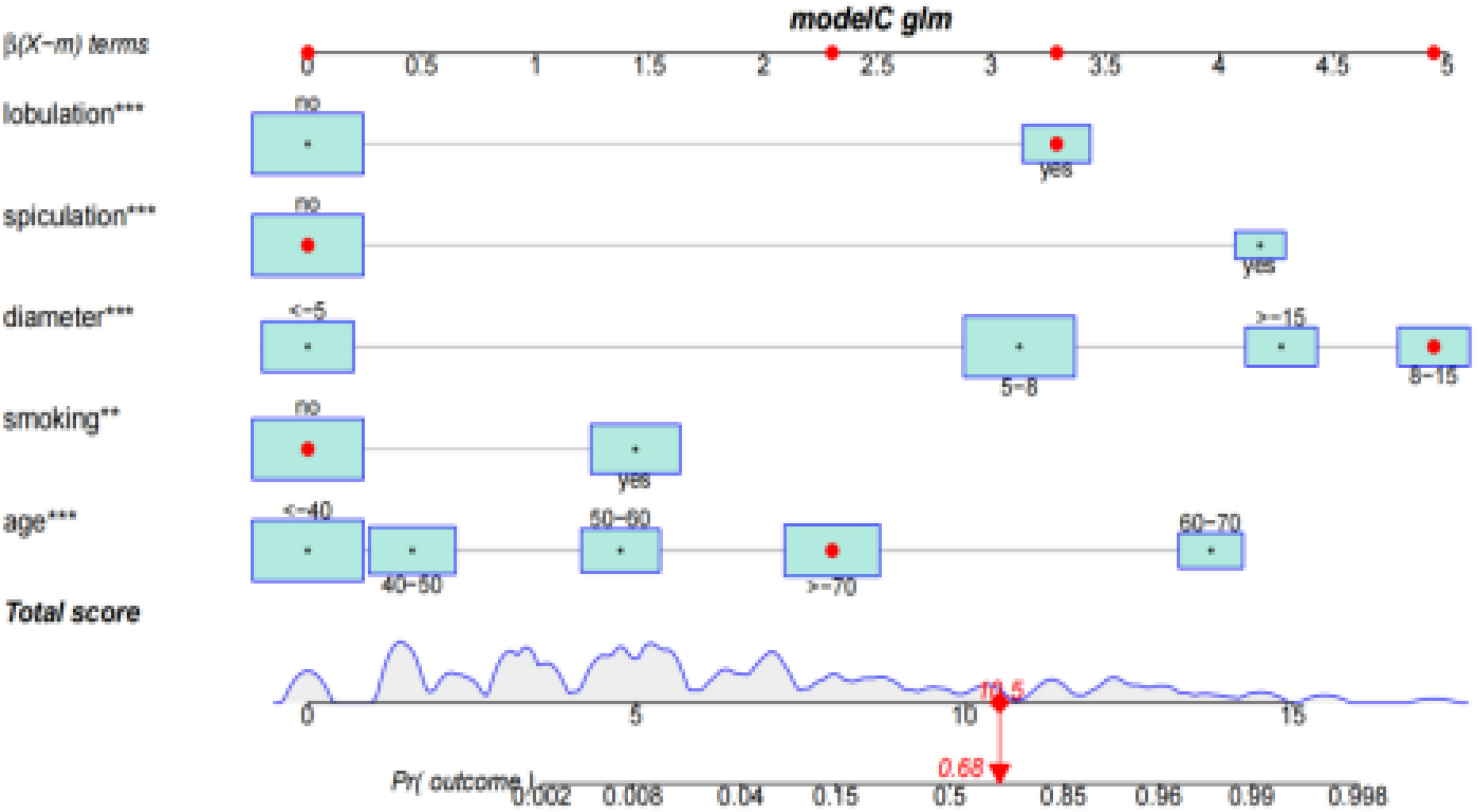
Visual predictive nomogram. *** denotes predictive value (i.e., the variable is a significant predictor).

Based on superior discrimination and calibration performance compared to the LASSO regression model, the logistic regression model was selected for nomogram construction. Five independent predictors from this model—lobulation, spiculation, smoking history, age, and nodule diameter—were incorporated to develop a visual predictive nomogram (**Figure 8**).

As illustrated in **Figure 7**, we selected a representative patient with a pulmonary nodule as an example. The characteristics were as follows: spiculation, negative; nodule diameter, 8–15 mm; smoking history, absent; lobulation, negative; age, ≥70 years. The total nomogram score was calculated as 10.5 points, corresponding to an estimated malignancy probability of approximately 68%.

## 4 Discussion

The widespread adoption of health check-ups and computed tomography (CT) has led to a yearly increase in the detection of solitary pulmonary nodules (SPNs) (1). However, the complex etiology of SPNs and the frequent absence of specific clinical signs (e.g., atelectasis, hilar enlargement, or pleural effusion) often result in false-positive reports in diagnostic evaluations (2). Therefore, accurately differentiating between benign and malignant SPNs remains a central challenge in clinical research. In this study, we developed and validated clinical prediction models for pulmonary nodule malignancy using logistic regression and LASSO regression (3). The logistic regression model identified five independent predictors: age (p = 0.04), smoking history (p = 0.02), nodule diameter (p = 0.01), lobulation (p = 0.02), and spiculation (p = 0.02). The LASSO regression model selected four predictors: spiculation, nodule diameter, carcinoembryonic antigen, and smoking history. Comparative performance analysis revealed that the logistic regression model outperformed the LASSO model in both discrimination and calibration. Consequently, a visual nomogram was constructed based on the logistic regression model results.

In this study, age and smoking history were identified as independent predictors of pulmonary nodule malignancy. These factors are not only integrated as key parameters in established international risk prediction models (e.g., Mayo, Brock, Herder, PanCan) but also exert their influence through molecular biological, epidemiological, and radiological pathways (4, 5).From a molecular carcinogenic perspective, smoking exposure exceeding 20 pack-years chronically exposes bronchial epithelium to over 60 class I carcinogens, including tar, nitrosamines, and polycyclic aromatic hydrocarbons (6). This impairs DNA damage repair systems (e.g., involving ERCC1, XRCC1, OGG1) and increases mutation accumulation rates in driver genes such as KRAS, TP53, EGFR, and STK11 by 5–10 fold (7), thereby accelerating malignant transformation. Concurrently, smoking-induced chronic airway inflammation, mediated by elevated IL-1β, IL-8, and TNF-α, promotes collagen deposition and stromal remodeling (8). This pathological microenvironment facilitates the development of malignant radiological features, including spiculation, lobulation, air bronchograms, pleural indentation, and vascular convergence on CT imaging (9).Malignancy probability exhibits an exponential increase with age. The proportion of malignant nodules is approximately 5% in individuals aged 40–49, rising to 15% (50–59 years), 35% (60–69 years), and exceeding 60–80% in those aged ≥70 years. This trend is associated with telomere shortening, epigenetic drift, immune senescence (e.g., reduced CD8+ T cells, PD-L1 upregulation), and cumulative environmental exposures such as secondhand smoke and air pollution. When advanced age coexists with heavy smoking, even nodules measuring 5–7 mm in diameter may confer a higher malignancy risk than larger nodules (e.g., >8 mm) (10).Given this evidence, the 2023 Chinese expert consensus recommends that individuals aged ≥50 years with a ≥20 pack-year smoking history and newly detected solid nodules undergo follow-up low-dose CT within three months, or proceed directly to PET−CT, electromagnetic navigation bronchoscopy, or thoracoscopic resection, to avoid missing curative treatment opportunities for early-stage lung cancer (11).

Our study further confirmed that nodule diameter and serum CEA level are significant predictors of SPN malignancy. The status of these two factors as “dual core” indicators in risk stratification is well established, as they reflect complementary dimensions of malignancy risk—nodule diameter provides a macroscopic, dose-response relationship, whereas CEA offers molecular-level early warning. Macroscopically, nodule diameter directly correlates with malignancy probability in a dose-dependent manner. For solid nodules, the malignancy rate is <1% for diameters <5 mm, 2–6% for 6–8 mm, 15–20% for >8 mm, and >50% for >20 mm (12). For subsolid nodules, the risk of invasive adenocarcinoma increases to approximately 60% when the pure ground-glass component measures ≥15 mm, and to 75% when the solid component reaches ≥6 mm. Moreover, about 83% of nodules with a volume doubling time <400 days are ultimately confirmed as malignant. At the molecular level, CEA serves as an early biomarker of malignant potential. The normal reference range is <3.4 ng/mL for non-smokers and <5.0 ng/mL for smokers. A CEA level >5 ng/mL yields a sensitivity of 40–60% and specificity of 80–90% for malignancy (13); when >10 ng/mL, specificity rises to 95%. A dynamic increase with a doubling time <6 months or a persistent linear rise is highly suggestive of invasive adenocarcinoma and is often associated with aggressive histologic features such as micropapillary/solid subtypes, STAS, and vascular invasion. When combined, a “diameter ≥8 mm + CEA ≥5 ng/mL” raises the probability of malignancy to approximately 63% in high-risk populations. Accordingly, Chinese guidelines based on data from 12,468 cases (2023 edition) recommend proceeding directly to PET−CT or electromagnetic navigation biopsy without an initial 3−month follow-up, an approach reported to increase early lung cancer detection by 22% (14). Similarly, in the Mayo model, nodule diameter contributes 35% and CEA 12% of the predictive weight; their combination elevates the AUC from 0.82 to 0.91, a result consistent with our findings.

In non-contrast chest CT, lobulation and spiculation are recognized as the “imaging dual markers” for predicting lung-nodule malignancy because they together constitute a quantifiable and reproducible system for assessing malignant risk across four dimensions: anatomical structure, pathological mechanism, molecular biology, and evidence-based data (15).First, in terms of anatomical-imaging correlation, spiculation presents as radial or brush-like projections from the nodule margin. Its pathological basis lies in the infiltrative growth of tumor cells along interlobular septa, perivascular-perilymphatic sheaths, and subpleural interstitium, which stimulates fibroblasts to produce abundant type III collagen and α-SMA-positive myofibroblasts, forming “tentacle-like” structures that extend into the surrounding lung parenchyma (16). High-resolution CT (≤1 mm slice thickness) clearly depicts these fine filamentous shadows (<2 mm), and multiplanar reconstruction (MPR) and minimum-intensity projection (MinIP) further reduce false-positive rates. Pooled data from three major international cohorts-NELSON, Lung-RADS, and PanCan—show that spiculation has a high positive predictive value (88–92%) for malignancy. Nodules with spiculation carry a 5.5-fold higher malignancy probability than those with smooth margins. In subsolid nodules, the presence of spiculation is also significantly associated with a solid−component ratio >50%, micropapillary/solid subtypes, and STAS (spread through air spaces), indicating higher-stage disease and earlier metastatic potential. Second, lobulation is characterized by irregular wavy or “potato-like” notches along the nodule contour. Its pathological mechanism is anisotropic tumor growth constrained by the elastic resistance of bronchi, pulmonary arteries, and interlobular septa (17). Deep lobulation (depth >3 mm) often corresponds to tumors that have breached alveolar walls and invaded adjacent lobules. Logistic regression confirms that lobulation is an independent risk factor for malignant solitary pulmonary nodules (OR = 3.3–4.5, 95% CI 2.6–7.1). The Danish Lung Cancer Screening Trial (n = 213) indicated that lobulated or notched margins raise the likelihood of malignancy from a baseline of about 15% to over 70%, with invasive adenocarcinoma accounting for 82% of cases when ≥3 lobulations are present (18, 19).When lobulation and spiculation occur together, they exert a synergistic effect in statistical models. A multicenter retrospective Chinese study (n = 1247) showed that malignancy probability rose from 50–60% for a single sign to 86% for the combination (20). The 2023 Fleischner Society guidelines list this combination as an imaging trigger for “immediate entry into further diagnostic procedures.” Decision-tree and random-forest models further confirm that, even after incorporating variables such as density, diameter, and location, “lobulation + spiculation” can still elevate the AUC to 0.746–0.783, with sensitivity of 91% and specificity of 78%.In clinical pathways, once this combination is detected on thin-slice CT, the “3A process” is initiated: AI−assisted volume measurement (to assess doubling time), advanced PET-CT (SUVmax ≥ 2.5), and navigational biopsy (electromagnetic navigation or radial EBUS) (21, 22). This reduces the average diagnostic time for early lung cancer from the traditional 90 days to 21 days, increases the resection rate for stage IA from 62% to 88%, and correspondingly raises the five-year survival rate by 12–15% (23, 24).Therefore, lobulation and spiculation are not only morphological codes in macroscopic imaging but also a visual window into microscopic tumor-host interactions. Their combined interpretation has become an indispensable core element in chest CT screening, follow-up, and surgical decision-making (25, 26).

Despite these findings, several limitations of this study should be acknowledged. First, due to its retrospective design, precise data for certain clinical variables — such as volumetric nodule growth rate and specific anatomical location — were not consistently available. Second, the limited sample size may constrain the statistical power of the analysis. Future research should expand the cohort and collect more comprehensive data to refine and retrain the current analytical framework, thereby improving the stability and reliability of the findings. Furthermore, this study did not perform in−depth stratified or multi−subgroup analyzes, and potential influences from various subgroup factors were not fully considered. Subsequent studies could build on this work by conducting more detailed stratification and subgroup explorations, which would help to better elucidate interactions among factors, refine the results, and offer more tailored guidance for clinical practice. While internal validation through random splitting and bootstrapping provides initial evidence of model stability, we recognize that external validation using independent multicenter data is essential to confirm transportability to diverse clinical settings. This was not feasible in the current study due to data availability constraints and single-center recruitment. Prospective multicenter external validation is planned as the immediate next step to assess model performance in different populations, imaging protocols, and prevalence settings before clinical implementation.

## Data Availability

The original contributions presented in the study are included in the article/**Supplementary Material**. Further inquiries can be directed to the corresponding author.
