# Supplementary material for: Construction and validation of a prediction model for malignant pulmonary nodules based on imaging, demographic, and laboratory features
Source: Front Oncol. 2026 Jul 27;16:1774574. doi: 10.3389/fonc.2026.1774574 (PMC13453707; doi:10.3389/fonc.2026.1774574)
Supplement: Supplementary file 2 [file Table1.docx]

| Method | AUC (95% CI) | Sensitivity | Specificity | PPV | NPV |
| --- | --- | --- | --- | --- | --- |
| Original dataset (3.5:1) | 0.874 | 0.828 | 0.744 | 0.804 | 0.783 |
| Random undersampling | 0.825 | 0.781 | 0.766 | 0.724 | 0.706 |
| SMOTE | 0.841 | 0.752 | 0.723 | 0.737 | 0.684 |
| Cost-sensitive | 0.804 | 0.747 | 0.735 | 0.732 | 0.735 |

Supplementary Table 1. Sensitivity analyses addressing the benign-to-malignant ratio of 3.5:1 demonstrated consistent model performance across random undersampling, SMOTE, and cost-sensitive learning approaches.
