# Supplementary material for: Construction and validation of a prediction model for malignant pulmonary nodules based on imaging, demographic, and laboratory features
Source: Front Oncol. 2026 Jul 27;16:1774574. doi: 10.3389/fonc.2026.1774574 (PMC13453707; doi:10.3389/fonc.2026.1774574)
Supplement: Supplementary file 3 [file DataSheet1.docx]

Supplementary Figure 1. Study flowchart
